# Correction: Development and validation of a model for predicting incident type 2 diabetes using quantitative clinical data and a Bayesian logistic model: A nationwide cohort and modeling study

**DOI:** 10.1371/journal.pmed.1004217

**Published:** 2023-03-30

**Authors:** Lua Wilkinson, Nengjun Yi, Tapan Mehta, Suzanne Judd, W. Timothy Garvey

The authors developed the model coefficients, which included the following variables and units: Blood Glucose (mg/dL), HDL cholesterol (mg/dL), Triglycerides (mg/dL) BMI (kg/m^2), blood pressure (mmHg), with an alternative model including waist circumference (cm). In the published article, however, these were converted to SI units. This was done without changing the coefficients of the model (i.e., equation) as listed in S2 Table.

To use the model correctly using the coefficients published, the user must input variables using conventional units, rather than the units published.
