# Supplementary material for: Development and validation of a model for predicting incident type 2 diabetes using quantitative clinical data and a Bayesian logistic model: A nationwide cohort and modeling study
Source: PLoS Med. 2020 Aug 7;17(8):e1003232. doi: 10.1371/journal.pmed.1003232 (PMC7413417; doi:10.1371/journal.pmed.1003232)
Supplement: S1 Table — (DOCX) [file pmed.1003232.s002.docx]

**Table S1.** External evaluations for Bayesian logistic model and four alternative methods.

|  | BhGLM | Lasso | Generalized additive model | Random forests | Support vector machine |
| --- | --- | --- | --- | --- | --- |
| AUC | 0.846 | 0.845 | 0.844 | 0.827 | 0.742 |
| MSE | 0.074 | 0.072 | 0.075 | 0.082 | 0.076 |
| Misclassification | 0.090 | 0.090 | 0.094 | 0.105 | 0.091 |

1. Gareth James, Daniela Witten, Trevor Hastie, Robert Tibshirani 2013 An Introduction to Statistical Learning, with Application in R. Springer New York.
