# Supplementary material for: Development and validation of a model for predicting incident type 2 diabetes using quantitative clinical data and a Bayesian logistic model: A nationwide cohort and modeling study
Source: PLoS Med. 2020 Aug 7;17(8):e1003232. doi: 10.1371/journal.pmed.1003232 (PMC7413417; doi:10.1371/journal.pmed.1003232)
Supplement: S1 Text — (DOCX) [file pmed.1003232.s004.docx]

**S1. Comparison with alternative methods**

We compared Bayesian logistic regression with four alternative methods: lasso, generalized additive model, random forests, and support vector machine^1^. Lasso is a penalized regression with L_1_ penalty, and can estimate the coefficients of irrelevant predictors exactly to zero. Generalized additive models (GAMs) provide a general framework for extending a standard linear model by allowing non-linear functions of the variables, while maintaining additivity. Random forest is a tree-based method, involving stratifying or segmenting the predictor space into a number of simple regions and making a prediction for a given observation using the mean or the mode of the training observations in the region to which it belongs. Support vector machine is a machine learning approach using kernels. We carried out these four alternative methods using R packages, glmnet, mgcv, randomForest, and kernlab, respectively.

We fit a predictive model using REGARDS and then evaluated the predictive performance in ARIC. As shown in Table S1, Bayesian logistic model provides better or similar prediction than these alternative methods. In addition, the logistic regression has advantages of being easily understood and clinically used.
