# Supplementary material for: Development and validation of a model for predicting incident type 2 diabetes using quantitative clinical data and a Bayesian logistic model: A nationwide cohort and modeling study
Source: PLoS Med. 2020 Aug 7;17(8):e1003232. doi: 10.1371/journal.pmed.1003232 (PMC7413417; doi:10.1371/journal.pmed.1003232)
Supplement: S2 Text — (DOCX) [file pmed.1003232.s005.docx]

**S2. Calculating the predictive risk probabilities of incident T2D**

The fitted main-effect logistic regression using REGARDS can be expressed as the following formula:

Pr (T2D) = logit^-1^ (-8.464 -0.014*Age + 0.053*BMI + 0.006*SBP

+ 0.003*DBP + 0.062*BG – 0.018*HDL + 0.001*TG

– 0.084*Sex – 0.466*Race)

The numbers in this formula are the estimated intercept and coefficients. The right side presents the probability of incident T2D for any individual; the function, logit^-1^ (x),

equals exp(x) / [1 + exp(x)]; Age, BMI, SBP, DBP, BG, HDL, and TG are the values of

age, BMI, systolic blood pressure, diastolic blood pressure, blood glucose, HDL-c, and

triglycerides, respectively, for the individual; Sex equals 1 for male and 0 for female,

and Race equals 1 for white and 0 for black. Thus, we can calculate the predictive risk

probabilities for any patients using their personal values.
