# Supplementary material for: Development and validation of a model for predicting incident type 2 diabetes using quantitative clinical data and a Bayesian logistic model: A nationwide cohort and modeling study
Source: PLoS Med. 2020 Aug 7;17(8):e1003232. doi: 10.1371/journal.pmed.1003232 (PMC7413417; doi:10.1371/journal.pmed.1003232)
Supplement: Attachment — Submitted filename: Response to Reviewers.docx [file pmed.1003232.s006.docx]

June 8, 2020

Dear PLOS Editors and Reviewers,

Thank you very much for reviewing our manuscript "Development and Validation of a Model for Predicting Incident Type-2 Diabetes Using Quantitative Clinical Data and a Bayesian Logistic Model: A Nationwide Cohort and Modelling Study" (PMEDICINE-D-20-00156R1) for consideration at PLOS Medicine.

We have read through all reviews and have implemented the comments and suggestions and wish to submit a revised version of the manuscript for further consideration. We thank the editors and reviewers for these comments which have led to an improved manuscript. Changes in the initial version are highlighted by track changes for clarity. Additionally, below we provide a point-by-point response explaining how we addressed each comment and suggestion.

We look forward to hearing about the outcome of your assessment.

Sincerely,

On behalf of all co-authors,

Lua Wilkinson, PhD, MA, RD

-----------------------------------------------------------
**Requests from the editors**:
Please revise your title according to PLOS Medicine's style. Your title must be nondeclarative and not a question. It should begin with main concept if possible. "Effect of" should be used only if causality can be inferred, i.e., for an RCT. Please place the study design ("A randomized controlled trial," "A retrospective study," "A modelling study," etc.) in the subtitle (ie, after a colon).

RESPONSE: The title has been changed to “Predicting Incident Diabetes Using Quantitative Clinical Data and a Bayesian Logistic Model for Cardiometabolic Disease Staging: A Nationwide Cohort and Modelling Study”

Abstract
a) Please provide brief participant demographics from both cohorts.

RESPONSE: We have added: 33% black Americans in REGARDS, 19% for ARIC.
b) If you meant T2D, please do replace instances of “diabetes” with Type 2 diabetes or T2D.

RESPONSE: We have now consistently referred to Type 2 Diabetes Mellitus as T2D throughout.
c) Last sentence of the methods and findings section should be a limitation of your study design/methodology.

RESPONSE: We have now expanded the discussion on limitations to include, for example limited generalizability of the REGARDS sample to black and white, older Americans, and the lack of time-to-diagnosis for T2D.”

Conclusions
a). Please start this section with “our results show” or similar.

RESPONSE: We have changed this phrase to “Our results show….”
b). Please avoid overreaching conclusions and use of adjectives such as “superior” to describe the predictive model

RESPONSE: Removed superior and changed to ‘improved’ only when accurate, and directly comparing to other models.

Author Summary
a). At this stage, we ask that you include a short, non-technical Author Summary of your research to make findings accessible to a wide audience that includes both scientists and non-scientists. The Author Summary should immediately follow the Abstract in your revised manuscript. This text is subject to editorial change and should be distinct from the scientific abstract. Please see our author guidelines for more information: <https://journals.plos.org/plosmedicine/s/revising-your-manuscript#loc-author-summary>

RESPONSE: Author summary has been added.

Prospective analysis plan
a). Did your study have a prospective protocol or analysis plan? Please state this (either way) early in the Methods section.

RESPONSE: Yes, we have included the TRIPOD checklist and page numbers.
b) If a prospective analysis plan (from your funding proposal, IRB or other ethics committee submission, study protocol, or other planning document written before analyzing the data) was used in designing the study, please include the relevant prospectively written document with your revised manuscript as a Supporting Information file to be published alongside your study, and cite it in the Methods section. A legend for this file should be included at the end of your manuscript. If no such document exists, please make sure that the Methods section transparently describes when analyses were planned, and when/why any data-driven changes to analyses took place.

RESPONSE: A prospective analysis plan was indeed drafted and was approved prior to analysis by the REGARDS committee, which additionally approved the manuscript in reference to the prospective analysis plan. These plans are attached. We did not add a sentence in the manuscript as this has not been standardly done with REGARDS published articles in the past.

c) In either case, changes in the analysis-- including those made in response to peer review comments-- should be identified as such in the Methods section of the paper, with rationale.

RESPONSE: All changes are listed in responses below.
d) Please ensure that the study is reported according to TRIPOD guideline, and include the completed checklist as Supporting Information. When completing the checklist, please use section and paragraph numbers, rather than page numbers. Please add the following statement, or similar, to the Methods: "This study is reported as per the Transparent Reporting of a Multivariable Prediction Model for Individual Prognosis or Diagnosis guideline (S1 Checklist)."

RESPONSE: This has not been standardly done for REGARDS analysis, however, we have included the checklist and requested sentence in a new paragraph starting out the Methods section.

Introduction
a) Could you add a reference at lines 67-68?

RESPONSE: Added – Centers for Disease Control and Prevention. National Diabetes Statistics Report, 2014 Estimates of Diabetes and Its Burden in the Epidemiologic estimation methods [Internet]. US Department of Health and Human Services. 2014 [cited 2017 Jun 21].

b) Could you please add a space between the text and reference brackets throughout, followed by a full stop. RESPONSE: Added throughout
c) Please introduce NHANES on first view.  RESPONSE: Added
d) Line 105- please introduce HDL. RESPONSE: Added

Methods
a) Line 130- please can you add United States here? RESPONSE: Added
b) Please provide the full name of the ethics committee that approved the protocol(s).

RESPONSE: Added

c) Line 142- it would be useful to have a bit more information regarding what analyses/measurements were carried out even if brief. You may wish to add a citation in addition, if this has been previously published.

RESPONSE: In the methods section, we explain, ”Analysis of blood specimens, physical measurements, and urine was performed using standardized methods. Participants were asked to fast for 10-12h before the visit (n = 9,332). Those who did not fast (n = 1,440) or have no information on fasting (n = 1,271) were included as non-fasters. DiabetesT2D was defined by having a fasting blood glucose level ≥126mg/dL, a non-fasting blood glucose ≥ 200mg/dL, self-reported diabetesT2D or on diabetes medication. Race was defined by self-report as black or white. Standardized blood pressure was taken twice in-home and calculated as the average of the two measurements. Lipids were assayed using either the fasting or non-fasting sample.” Citations are also provided regarding operational features of the cohort studies. Readers can find more in depth description of cohort study operations and measurements within the cited references.

Bibliography

a) Please format your bibliography to Vancouver style. RESPONSE: Changed to Vancouver style.

 **Comments from the reviewers:**

**Reviewer #1:**

a) Is the REGARDS dataset (2003-2007), collected for stroke patients, representative of the wider population in order to analyse T2DM incidence? The population was not designed to assess diabetes.
How do the baseline population characteristics compare to the wider population, in order to extrapolate results?

RESPONSE: The Reviewer has raised an important and germane question. REGARDS recruitment was a national scientific sample based on the regional density of stroke. The sampling excluded prior stroke and was intended to be population based with one exception, the goal was equal numbers of blacks and white and men and women. Within this construct, the sample is well suited to assess incident diabetes, CVD events, and other cardiometabolic disease manifestations. The nature of the data collected was well suited to assess risk for both metabolic and vascular disease given the extensive overlap in the risk factors for diabetes and stroke. While this is an assumption, the risk factors predisposing to this regionally enriched recruitment is highly likely to be would operative in individuals living in US regions less densely sampled in REGARDS. It is this no surprise that the model was well validated in a second cohort, the ARIC study.

b) "External validation was performed using 9,710 participants from Atherosclerotic Risk in Communities (ARIC) (1987-1989), observed through 1996-1998."Is this population an unbiased sampling frame for T2DM?

RESPONSE: The Atherosclerosis Risk in Communities Study (ARIC), sponsored by the National Heart, Lung, and Blood Institute (NHLBI) is a prospective epidemiologic study conducted in four diverse U.S. communities. ARIC is designed to investigate the causes of atherosclerosis and its clinical outcomes, and variation in cardiovascular risk factors, medical care, and disease by race, gender, location, and date. The ARIC study emphasized substantial recruitment of Blacks, which made this an attractive cohort for our validation study. ARIC has been widely used for T2D research and a diabetes calculator has been developed on this sample.

https://care.diabetesjournals.org/content/28/8/2013.long

To date, the ARIC study has published over 2,000 articles in peer-reviewed journals.

c) "...Atherosclerotic Risk in Communities (ARIC)[11] cohorts to predict incident diabetes with specificity for sex and race. CMDS was developed using quantitative measures of metabolic syndrome traits (i.e., ATP-III criteria)[12], with the limitation that these cohorts were not designed as nationally representative." Are the authors referring to the same cohort that they are using for their external validation?

RESPONSE: Yes

d) How did the authors cope with any potential changes in definition or testing methods of T2DM over time between baseline and follow up?

RESPONSE: Both the WHO and an ADA Expert Committee established in 1998 that a fasting glucose concentration of > or = 126 mg/dl (7.0 mmol/L) constitutes a diagnostic criterion for diabetes, and retained the criterion of random glucose > or = 200 mg/dl (11.1 mol/L) which had been in effect since 1979. In our data analyses, diabetes has been operationally defined using these criteria across the duration of the cohort studies.

e) The authors use a Bayesian logistic model using full-information continuous predictors. The novelty of this research piece is in its' application of continuous variables and interactions.
The method of Bayesian logistic modelling, and the means of measuring and comparing performance, seem appropriate given the context of this research question.

RESPONSE: We thank the Reviewer for this affirmation.

f) "Between the first and second visit, 5,713 individuals died, and 8,532 withdrew from further follow-up, leaving a population of 15,938 with follow-up data available."
What was cause of death (i.e. were any T2DM related?)? Were reasons for withdrawing from follow up provided (in order to understand if this missing data can be considered to be missing at random)? –

RESPONSE: We do not know reasons for dropout, and added the sentence, “While the reasons for withdrawing from follow-up is unknown, previous work in this population shows that missing data do not change exposure outcome relationships in a study such as REGARDS (30).”, with the appropriate citation.

g) "Diabetes was defined by having a fasting blood glucose level ≥126mg/dL, a non-fasting blood glucose ≥ 200mg/dL, self-reported diabetes or on diabetes medication". Is there a risk of misclassification for cases of self diagnosed T2DM? This is part of the published ARIC protocol, but what is the potential impact in this setting?

RESPONSE: Self-reported diagnosis of T2D was used as an adjunct to blood measurements or being on a diabetes medication. While this may include some reporting bias, we are unaware of self-reporting disease biasing a person’s response. As this was used to complement blood measurements, we examined those who had self-reported T2D and compared these with those also on medication, as well as blood measurements; in all, there were 12 individuals who reported being diagnosed with diabetes and reported not taking medication who also had a fasting blood glucose <= 126 mg/dL or non-fasting <=200mg/dL.

h) There are some grammatical errors in the text, for example the statistical methods section."Cauchy priors, which has advantage of providing"

RESPONSE: Thank you. We have re-reviewed all sections and fixed grammatical errors.

(i) Did the authors assess correlation between parameters in the model, and the effect this might have on the model outcome and interpretation?

RESPONSE: In the manuscript, we show the correlation between waist circumference and BMI (0.74). We changed the section title here to “Correlation of Parameters: Waist Circumference and BMI” and added an additional sentence in the revision: the correlation between SBP and DBP is 0.62, but others are low, < 0.2. As such, there was minimal or no influence of these correlations on the model.

(j) The authors provide a clear description of findings in the results section, aligned with informative tables and figures.

RESPONSE: The authors appreciate this comment

(k) "additional reason for greater predictive value is that the previous models did not include age.[19] "
Can the authors compare their model with previous models updated to include age (albeit dichotomously), in order to compare how much uplift in the model performance is down to the inclusion of this variable, and how much is due to the novel application of Bayesian modelling?

RESPONSE: We thank the Reviewer for this comment. We re-ran the Bayesian logistic model without ‘age’, and found that removing ‘age’ does not decrease the prediction: AUC = 0.788 in Regards and 0.845 in Aric.” Therefore, ‘age’ does not greatly increase the prediction, and this paragraph was taken out.

**Reviewer #2:**

This is a well-conducted study that developed a Bayesian logistic model using full-information continuous predictors to predict T2DM risk. This tool can be used for diabetes prevention efforts including weight loss therapy by allowing clinicians to target high risk individuals in a manner that could be used to optimize outcomes.
The authors developed the model in REGARDS and validated it in ARIC, two large national cohorts including both White and Black men and women. They compared the Bayesian method with several other predictive modeling methods, including lasso, generalized additive model, random forests, and support vector machine learning, and found that the Bayesian logistic model outperformed these alternative approaches.
They further compared the AUC from the Bayesian logistic model to the CMDS model, the Framingham and American Diabetes Association risk scores. The AUC for the Bayesian logistic model was superior at AUC 0.79 to other models or scores, such as the CMDS model (AUC 0.75), the Framingham scoring system (AUC 0.76) and American Diabetes Association scoring system (AUC 0.64).

RESPONSE: We thank the Reviewer for these very positive comments.

**Reviewer #3:**

(a) This manuscript conducted a Bayesian logistic model using the full-information continuous analysis with the nationally sampled data from white and black American adults to predict T2DM risk. The manuscript is well written and provides a powerful predictive tool to used for diabetes prevention.

RESPONSE: We appreciate the Reviewer’s affirmative comment

(b) There were some issues related to this manuscript. As the authors indicated that there were 8,532 participants withdrew from further follow-up in the REGARDS. Since that the REGARDS is a nationally sampled longitudinal survey, are there any differences between these 8,532 participants and the participants remained for the further analysis in the present study?

RESPONSE: We thank the Reviewer for making this excellent point which does represent a limitation that is now clearly stated in the revised manuscript. The text states: “Between the first and second REGARDS survey, 8,532 participants withdrew from further follow-up; upon inspecting demographic and metabolic profile (including the 8 traits examined) differences between these 8,532 participants and those that remained, only baseline BMI and diastolic blood pressure showed no significant difference. While the reasons for withdrawing from follow-up is unknown, previous work in this population shows that missing data do not change exposure outcome relationships in a study such as REGARDS (30).”

(c) Line 230, age (45-92) should be age (45-92 y). RESPONSE: This has been changed.

(d) In the Table A2, the min value of BMI, waist circumference, blood glucose, and HDL are extremely low. it is better to exclude these participants with outlier. Also, the unit (kg/m2) for BMI and cm for waist circumference should add in this table.

RESPONSE: Thank you. We considered this at length during the preliminary analysis, and decided to leave those with some of the extreme values as there were few and these were not leverage points such that they do not influence the fit.

(e) The mean age of the REGARDS survey is 63.12 years. Therefore, generalizability to younger population also requires caution.

RESPONSE. We thank the Reviewer again. Yes, we have now clearly indicated that the age of the subjects should be considered in interpreting the data, and we have added this as a limitation in the Discussion section.

_________________________________

Again, we thank the Editors and Reviewers for the constructive critiques, which has resulted in a clearer presentation of the data and an improved manuscript. We hope that our revision is now acceptable for publication.

Sincerely.

Lua Wilkinson, PhD, MA, RD
