# Supplementary material for: Development and validation of a model for predicting incident type 2 diabetes using quantitative clinical data and a Bayesian logistic model: A nationwide cohort and modeling study
Source: PLoS Med. 2020 Aug 7;17(8):e1003232. doi: 10.1371/journal.pmed.1003232 (PMC7413417; doi:10.1371/journal.pmed.1003232)
Supplement: Attachment — Submitted filename: FInal_revisions_RESPONSE.docx [file pmed.1003232.s007.docx]

Dear Editorial Board,        

Please see our responses to the formatting issues. Thank you,

Lua Wilkinson and co-authors

1. Please ensure that each supporting information file has a legend at the end of the manuscript file, after the Reference list.
   a. RESPONSE: This was double checked and file S1 TRIPOD checklist legend was added.

2. Figures 1 and 2 have text that looks smaller than 8 points. The legibility of type at sizes smaller than 8 points in the published PDF is frequently compromised. We cannot assume responsibility for the legibility of Figures in the published PDF if there is type smaller than 8 point in them. Please note that this problem will not be detected or corrected by the PACE tool. We recommend that you print figures out to get a better idea of the text legibility. If you are happy with the way they look, we will publish them with the font as is.
   a. RESPONSE: This was re-evaluated and re-uploaded with the correct font

3. Figure 2 currently contains more than ½ an inch of white space on any side. Please remove unnecessary white space, leaving about ⅛ inch of white space outside of the image. Once you have made your selection, go to Image → Crop to Selection. After cropping, always be sure to check the size.
   a. RESPONSE: This was cropped and re-uploaded.

- Figures 3 and 4 are acceptable for publication.
